# Gastroenteropancreatic neuroendocrine tumor of the accessory papilla of the duodenum: a case report

**DOI:** 10.1186/s40792-021-01241-4

**Published:** 2021-06-30

**Authors:** Kosei Takagi, Yuzo Umeda, Ryuichi Yoshida, Kazuhiro Yoshida, Kazuya Yasui, Hiroki Sato, Takahito Yagi, Toshiyoshi Fujiwara

**Affiliations:** Department of Gastroenterological Surgery, Okayama University Graduate School of Medicine, Dentistry, and Pharmaceutical Sciences, 2-5-1 Shikata-cho, Kita-ku, Okayama, 700-8558 Japan

**Keywords:** Accessory papilla of the duodenum, Neuroendocrine tumor, Carcinoid tumor

## Abstract

**Background:**

Contrary to the increasing incidence of gastroenteropancreatic neuroendocrine tumors (GEP-NETs), GEP-NETs of the accessory papilla of the duodenum are extremely rare. Furthermore, there have been no recommendations regarding the treatment strategy for GEP-NETs of the accessory papilla of the duodenum. We present a case of GEP-NET of the accessory papilla of the duodenum successfully treated with robotic pancreatoduodenectomy.

**Case presentation:**

A case of a 70-year-old complaining of no symptoms was diagnosed with GEP-NET of the accessory papilla of the duodenum. A 8-mm tumor was located at the submucosal layer with a biopsy demonstrating a neuroendocrine tumor grade 1. The patient underwent robotic pancreatoduodenectomy as curative resection for the tumor. The total operative time was 406 min with an estimated blood loss of 150 mL. The histological examination revealed a well-differentiated neuroendocrine tumor with low Ki-67 index (< 1%). In the posterior areas of the pancreas, the lymph node metastases were detected. The patient was followed up for 6 months with no recurrence postoperatively.

**Conclusions:**

Considering the potential risks of the lymph node metastases, the standard treatment strategy for GEP-NETs of the accessory papilla of the duodenum should be radical resection with pancreatoduodenectomy. Minimally invasive approach can be the alternative to the conventional open surgery.

## Background

Although the incidence of gastroenteropancreatic neuroendocrine tumors (GEP-NETs) has increased, GEP-NETs of the duodenum are rare [1, 2]. Furthermore, GEP-NETs of the accessory papilla of the duodenum are extremely rare. To date, there have been only nine cases of GEP-NETs of the accessory papilla of the duodenum reported in the English literature [3–11]. We hereby present a case of GEP-NET of the accessory papilla of the duodenum successfully treated with robotic pancreatoduodenectomy.

## Case presentation

**Fig. 1:**
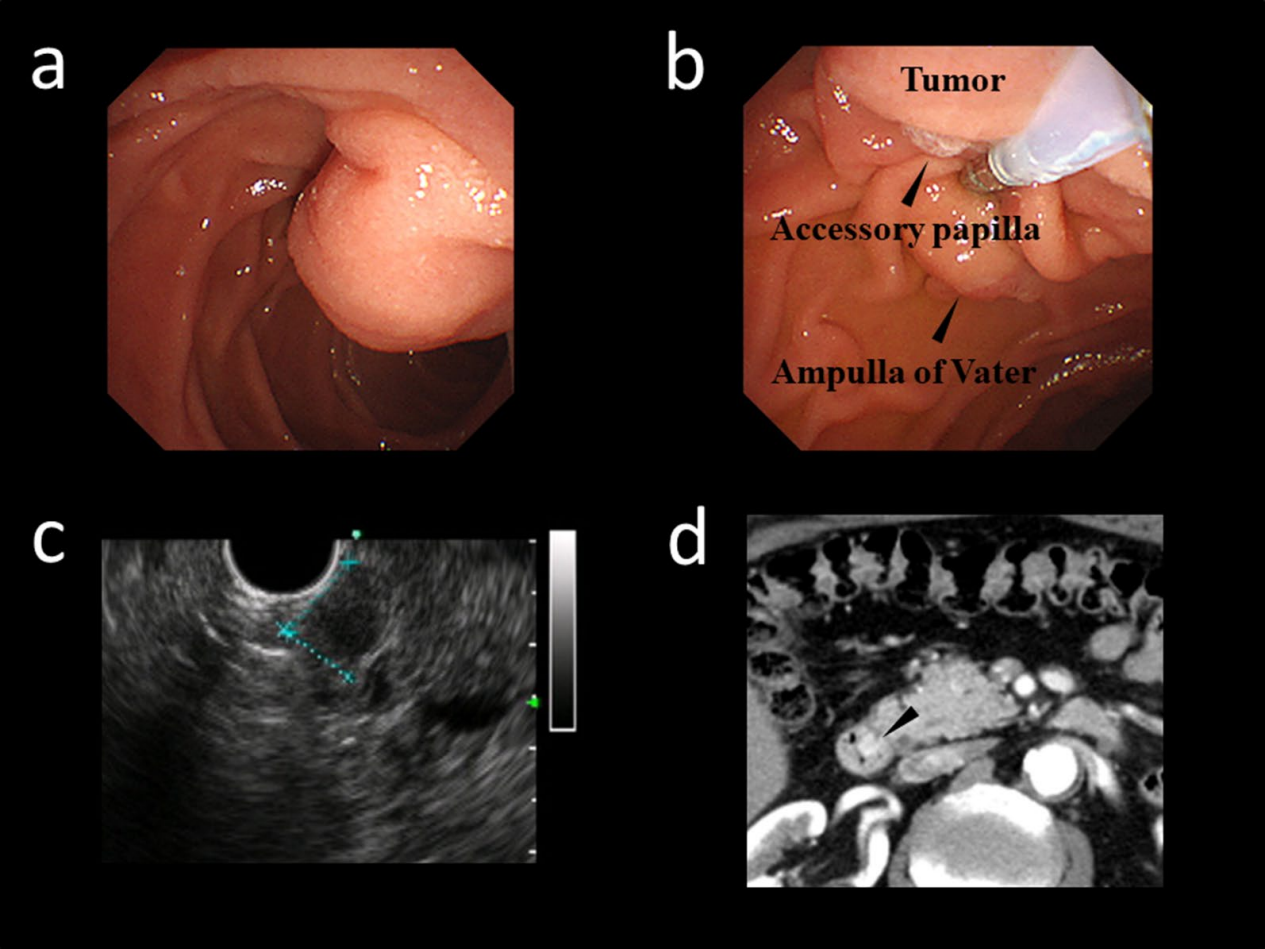
Endoscopic examination showed a 8-mm submucosal tumor at the accessory papilla in the second part of the duodenum (**a**). The relationship between the tumor, the accessory papilla and the ampulla of Vater is demonstrated (**b**). Endoscopic ultrasonography demonstrated hypoechoic mass in the submucosal layer (**c**). Computed tomography (CT) revealed the highly enhanced tumor in the early phase with no metastatic lesion including the lymph node and the liver (**d**)

A 70-year-old man was referred to our hospital for further examination of a tumor at the duodenum. Esophagogastroduodenoscopy identified a submucosal tumor at the accessory papilla in the second part of the duodenum (Fig. 1a, b). Endoscopic ultrasonography showed a 8-mm hypoechoic mass in the submucosal layer (Fig. 1c), and endoscopic biopsy demonstrated a well-differentiated neuroendocrine tumor with a low Ki-67 index suggesting grade 1 (G1). Computed tomography (CT) revealed a highly enhanced tumor in the early phase, showing neither apparent lymph node metastases nor distant metastases (Fig. 1d). Magnetic resonance imaging (MRI) also detected no lymph node and liver metastases.

Although there have been no recommendations regarding the treatment strategy for GEP-NETs of the accessory papilla of the duodenum, radical resection with pancreatoduodenectomy has been recommended as the first-line treatment for GEP-NETs of the ampulla of Vater due to relatively poor prognosis [12]. Furthermore, endoscopic intervention was considered to be difficult due to the potential risk of the proper muscular layer damage of the duodenum as well as location at the accessory papilla. Therefore robotic pancreatoduodenectomy with lymph node dissection was performed.

Our standardized protocol for robotic pancreatoduodenectomy was previously reported [13]. Following the kocherization of the duodenum, the jejunum and the stomach are divided. The hepatoduodenal ligament was dissected to perform hilar lymphadenectomy. After the transection of the pancreas on the superior mesenteric vein, the uncinate dissection was performed along the superior mesenteric artery. Thereafter the specimen was removed through the Pfannenstiel incision. The reconstruction included the pancreaticojejunostomy with interrupted two-layer modified Blumgart method, the hepaticojejunostomy with interrupted sutures, and the gastrojejunostomy with the robotic-sewn anastomosis.

**Fig. 2:**
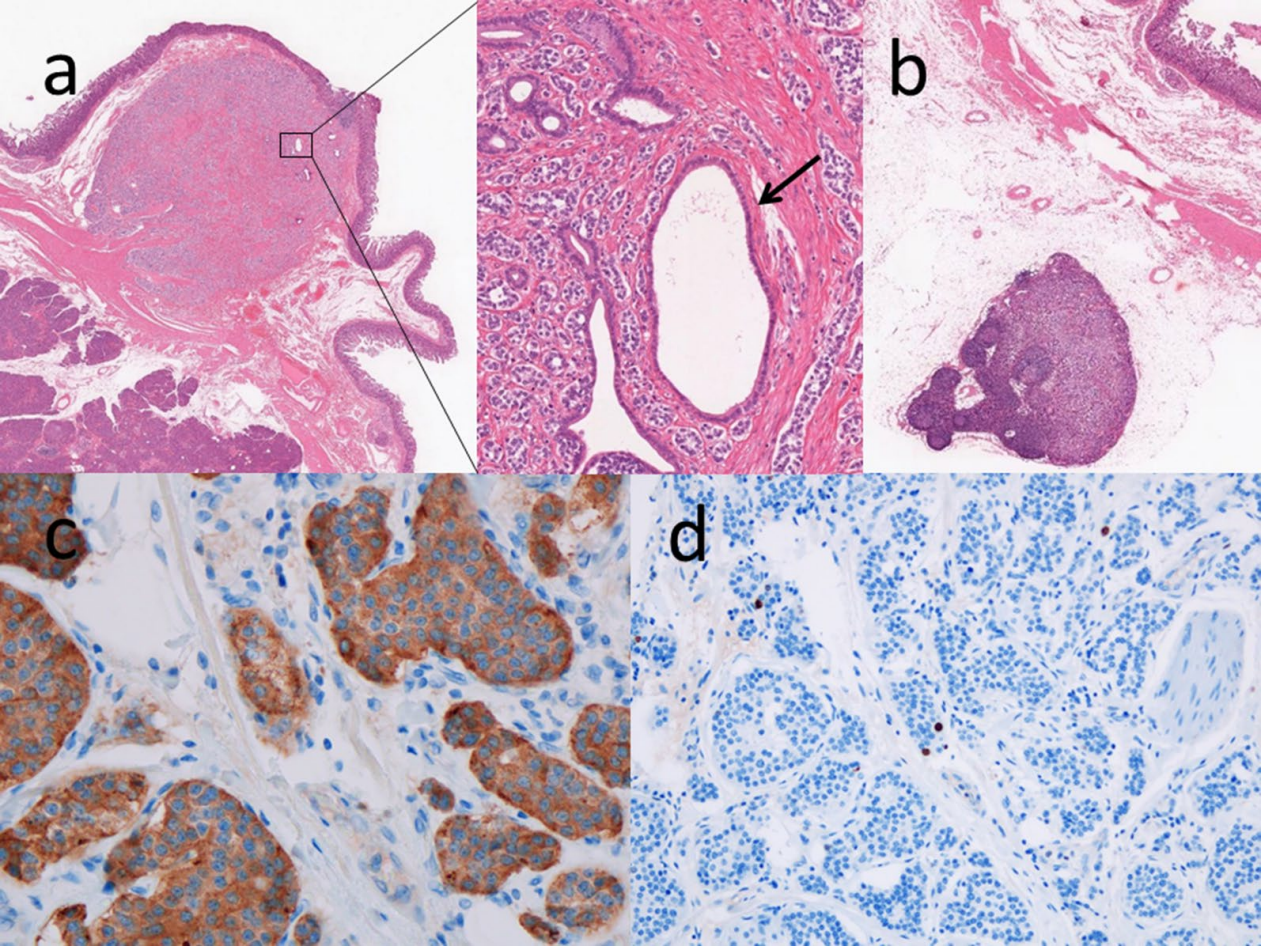
The tumor at the accessory papilla as the accessory pancreatic duct (arrow) is shown. Hematoxylin and eosin stain demonstrating nesting pattern of mostly uniform cells with central ovoid nucleus (**a**). The lymph node metastasis (**b**). Synaptophysin showing positive (**c**). Ki-67 index stain showing < 1% proliferation (**d**)

The total operative time was 406 min, and the estimated blood loss was 150 mL. Uneventful postoperative course including no complications was observed with the patient being discharged on postoperative day 8. The final histological diagnosis was neuroendocrine tumor G1 based on the findings of synaptophysin positive, chromogranin A positive, and Ki-67 index (< 1%) (Fig. 2). The presence of lymphovascular invasion was not found in the D2-40 immunostaining. Surprisingly, the lymph node metastases were confirmed at posterior areas of the pancreas (station 13). Regarding immunohistochemical staining of endocrine cells, the insulin-, gastrin- and somatostatin-immunoreactive endocrine cells were not detected.

The patient was followed up for 6 months without recurrence postoperatively.

## Discussion

To our knowledge, this is the first description on GEP-NETs of the accessory papilla of the duodenum with lymph node metastases successfully treated with robotic pancreatoduodenectomy. The present case indicates that a well-differentiated neuroendocrine tumor G1 with less than 1 cm at the accessory papilla of the duodenum could have the potential risks of the lymph node metastases. Therefore we suggest that radical surgical resection with pancreatoduodenectomy instead of local excision should be the first-line treatment option for GEP-NETs of the accessory papilla of the duodenum.

Contrary to the increasing number of GEP-NETs, GEP-NETs of the accessory papilla of the duodenum are very rare. The characteristics in reported cases of GEP-NETs of the accessory papilla of the duodenum are demonstrated in Table 1 [3–11]. The average tumor size was approximately 11 mm. Most of cases developed symptoms including pain, diarrhea, and weight loss. Regarding the type of intervention, five cases out of ten received local excision with surgically (*n* = 4) or endoscopically (*n* = 1). Although the margin of resected tumor was confirmed to be positive in a case with endoscopic intervention, no recurrences during the follow-up were observed in five cases undergoing local excision. In contrast, five cases underwent radical resection with pancreatoduodenectomy. The results of case series suggested the efficacy of radical resection for the tumors. Surprisingly, lymph node metastases at the resected regional areas were identified in four cases out of five. Therefore, it should be recognized that GEP-NETs of the accessory papilla of the duodenum could metastasize to the lymph nodes regardless of size of the tumor.

**Table 1:** Characteristics in reported cases of gastroenteropancreatic neuroendocrine tumors of the accessory papilla of the duodenum

Reports (year)	Age/sex	Symptom	Size (mm)	Treatment	Pathology	Outcome
Malone et al. (1985) [3]	46/M	Abdominal pain Anorexia Steatorrhea Wight loss	5–7.5	Local excision (Surgically)	Somatostatinoma	No recurrence, 16 months
Borobia et al. (2001) [4]	46/F	Diarrhea Steatorrhea	NA	Local excision (Surgically)	Neuroendocrine tumor	No recurrence, 3 years
Singh et al. (2003) [5]	35/F	Pancreatitis	10	Local excision Sphincteroplasty (surgically)	Carcinoid Negative margin	No recurrence, 6 months
Wang et al. (2005) [6]	50/M	Multiple melenas Polycythemia vera	9	Local excision (surgically)	Carcinoid	No recurrence, 3 years
Itoi et al. (2007) [7]	65/M	No	12	Local excision (endoscopically)	Invasive carcinoid tumor Positive margin	No recurrence, 4 years
Stömmer et al. (1987) [8]	56/F	Jaundice Wight loss	3	PD	Somatostatinoma No LNM	NA
Lowes et al. (1988) [9]	50/F	Abdominal pain Diarrhea Wight loss	12	PD	Somatostatinoma LNM (regional areas)	NA
Waisberg et al. (2006) [10]	57/F	Abdominal pain Diarrhea Weight loss	27	PD	Carcinoid (somatostatinoma) LNM (regional areas)	Death, POD 21
Kim et al. (2010) [11]	56/F	Abdominal pain	12	PD	Carcinoid Vascular invasion No perineural invasion LNM (regional areas)	NA
Our case	70/M	No	8	Robotic PD	Neuroendocrine tumor Well-differentiated (G1) No lymphovascular invasion LNM (regional areas)	No recurrence, 6 months

*M* male, *F* female, *PD* pancreatoduodenectomy, *LNM* lymph node metastasis, *NA* not available

With respect to treatment strategy, it suggests that pancreatoduodenectomy should be the first-line treatment for GEP-NETs of the accessory papilla of the duodenum. In the light of malignant potential, radical resection should be considered even for small primary tumors. Minimally invasive pancreatoduodenectomy can be optional given the safety and oncologic efficacy of minimally invasive surgery [14]. In contrast, surgical or endoscopic local excision should not be recommended as curative treatment, but can be an alternative. In addition, local excision with regional lymph node dissection or sentinel node dissection could be an option as less invasive surgical intervention. Prior to performing local excision, accurate radiographic evaluations are important for localizing tumors. CT and MRI are the most common modalities used for evaluation of the primary tumor and metastasis [15]. Moreover, somatostatin receptor scintigraphy has improved accuracy to detect smaller GEP-NETs [15]. Accordingly, local excision should be performed only in limited cases such as high-risk patients for surgery or patients with no lymph node metastases. However, further clinicopathological evidence including short-term and long-term outcomes on GEP-NETs of the accessory papilla of the duodenum is needed.

In conclusion, we present an extremely rare case of GEP-NET of the accessory papilla of the duodenum accompanied by lymph node metastases. Although carcinogenic mechanism of this tumor remains uncertain because of the limited reports, radical resection with pancreatoduodenectomy should be performed due to the potential risks of the lymph node metastases. Minimally invasive approach should be considered as the alternative to the conventional open surgery.

## Data Availability

Data supporting the conclusions are included in the article.

## Abbreviations

GEP-NET: Gastroenteropancreatic neuroendocrine tumor
CT: Computed tomography
MRI: Magnetic resonance imaging
